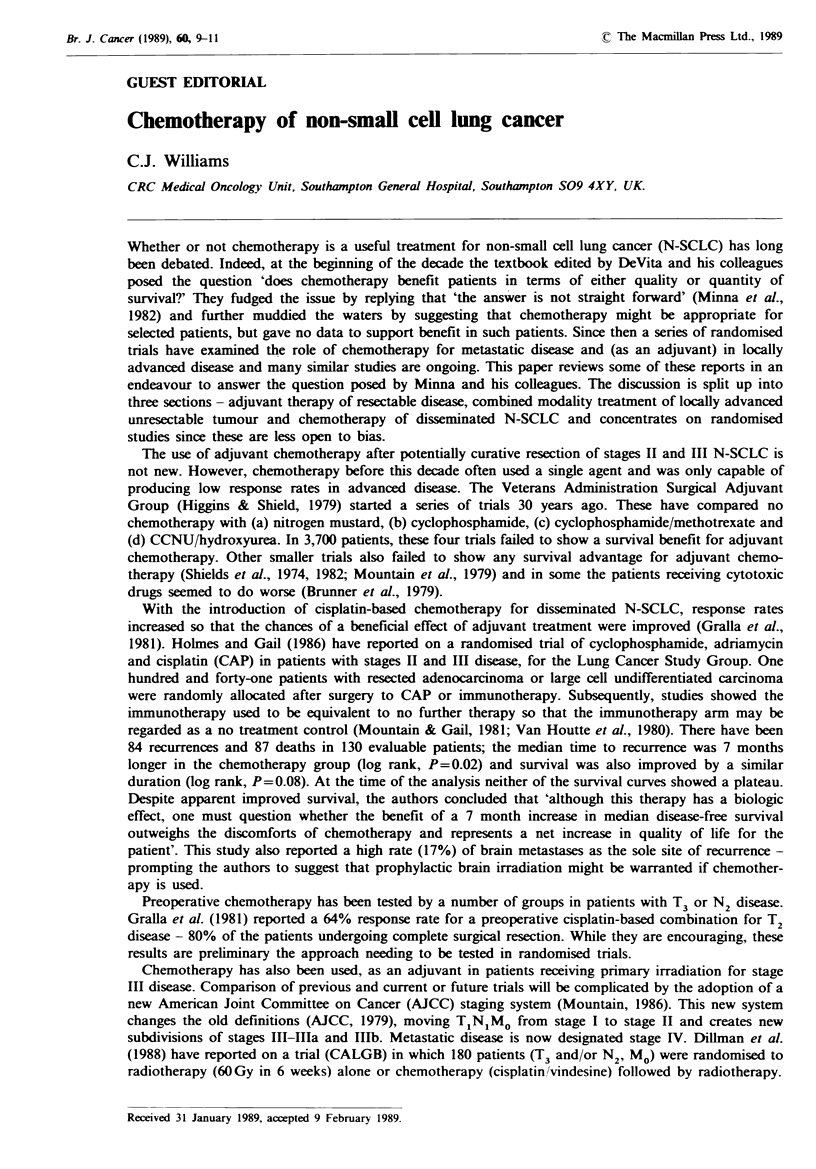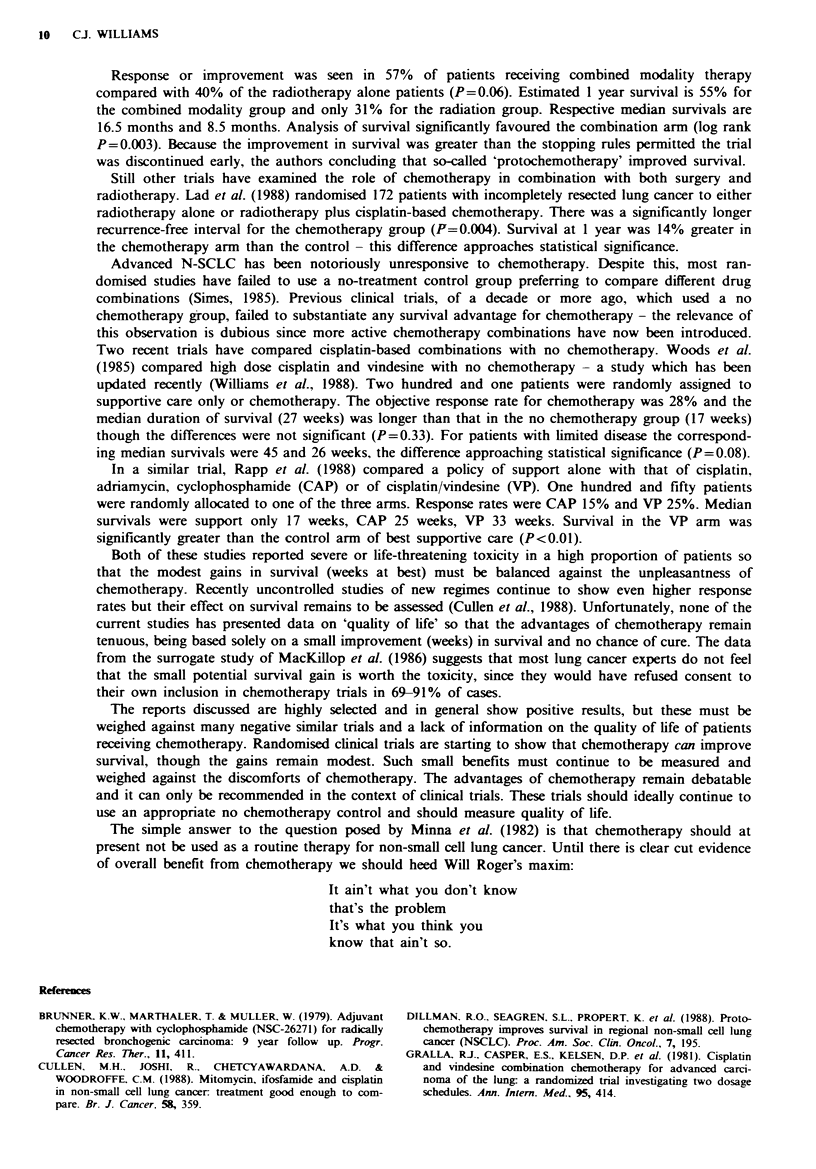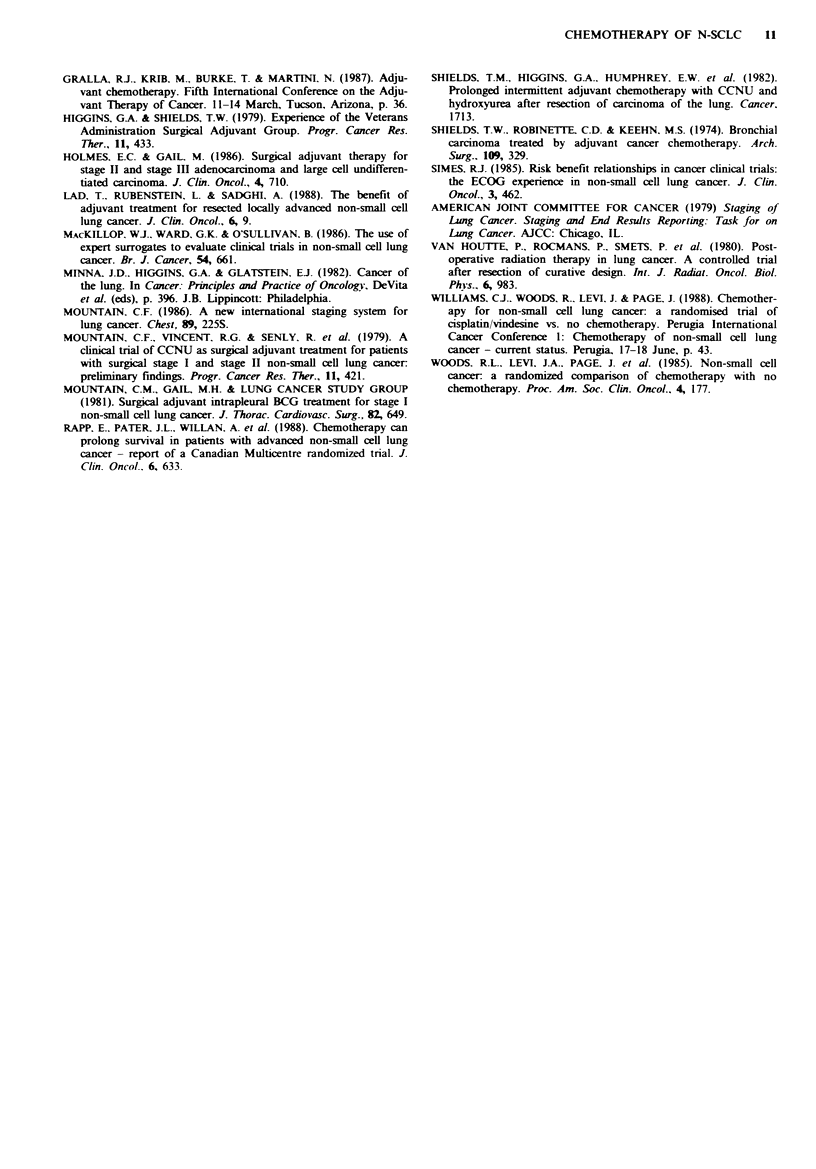# Chemotherapy of non-small cell lung cancer.

**DOI:** 10.1038/bjc.1989.209

**Published:** 1989-07

**Authors:** C. J. Williams

**Affiliations:** CRC Medical Oncology Unit, Southampton General Hospital, UK.


*Br. J. Cancer* (1989), 60, 9–11 © The Macmillan Press Ltd., 1989

**GUEST EDITORIAL**

### Chemotherapy of non-small cell lung cancer

C.J. Williams

*CRC Medical Oncology Unit, Southampton General Hospital, Southampton SO9 4XY, UK.*

Whether or not chemotherapy is a useful treatment for non-small cell lung cancer (N-SCLC) has long been debated. Indeed, at the beginning of the decade the textbook edited by DeVita and his colleagues posed the question 'does chemotherapy benefit patients in terms of either quality or quantity of survival?' They fudged the issue by replying that 'the answer is not straight forward' (Minna *et al.*, 1982) and further muddied the waters by suggesting that chemotherapy might be appropriate for selected patients, but gave no data to support benefit in such patients. Since then a series of randomised trials have examined the role of chemotherapy for metastatic disease and (as an adjuvant) in locally advanced disease and many similar studies are ongoing. This paper reviews some of these reports in an endeavour to answer the question posed by Minna and his colleagues. The discussion is split up into three sections – adjuvant therapy of resectable disease, combined modality treatment of locally advanced unresectable tumour and chemotherapy of disseminated N-SCLC and concentrates on randomised studies since these are less open to bias.

The use of adjuvant chemotherapy after potentially curative resection of stages II and III N-SCLC is not new. However, chemotherapy before this decade often used a single agent and was only capable of producing low response rates in advanced disease. The Veterans Administration Surgical Adjuvant Group (Higgins & Shield, 1979) started a series of trials 30 years ago. These have compared no chemotherapy with (a) nitrogen mustard, (b) cyclophosphamide, (c) cyclophosphamide/methotrexate and (d) CCNU/hydroxyurea. In 3,700 patients, these four trials failed to show a survival benefit for adjuvant chemotherapy. Other smaller trials also failed to show any survival advantage for adjuvant chemotherapy (Shields *et al.*, 1974, 1982; Mountain *et al.*, 1979) and in some the patients receiving cytotoxic drugs seemed to do worse (Brunner *et al.*, 1979).

With the introduction of cisplatin-based chemotherapy for disseminated N-SCLC, response rates increased so that the chances of a beneficial effect of adjuvant treatment were improved (Gralla *et al.*, 1981). Holmes and Gail (1986) have reported on a randomised trial of cyclophosphamide, adriamycin and cisplatin (CAP) in patients with stages II and III disease, for the Lung Cancer Study Group. One hundred and forty-one patients with resected adenocarcinoma or large cell undifferentiated carcinoma were randomly allocated after surgery to CAP or immunotherapy. Subsequently, studies showed the immunotherapy used to be equivalent to no further therapy so that the immunotherapy arm may be regarded as a no treatment control (Mountain & Gail, 1981; Van Houtte *et al.*, 1980). There have been 84 recurrences and 87 deaths in 130 evaluable patients; the median time to recurrence was 7 months longer in the chemotherapy group (log rank, $P=0.02$) and survival was also improved by a similar duration (log rank, $P=0.08$). At the time of the analysis neither of the survival curves showed a plateau. Despite apparent improved survival, the authors concluded that 'although this therapy has a biologic effect, one must question whether the benefit of a 7 month increase in median disease-free survival outweighs the discomforts of chemotherapy and represents a net increase in quality of life for the patient'. This study also reported a high rate (17%) of brain metastases as the sole site of recurrence – prompting the authors to suggest that prophylactic brain irradiation might be warranted if chemotherapy is used.

Preoperative chemotherapy has been tested by a number of groups in patients with $T_3$ or $N_2$ disease. Gralla *et al.* (1981) reported a 64% response rate for a preoperative cisplatin-based combination for $T_2$ disease – 80% of the patients undergoing complete surgical resection. While they are encouraging, these results are preliminary the approach needing to be tested in randomised trials.

Chemotherapy has also been used, as an adjuvant in patients receiving primary irradiation for stage III disease. Comparison of previous and current or future trials will be complicated by the adoption of a new American Joint Committee on Cancer (AJCC) staging system (Mountain, 1986). This new system changes the old definitions (AJCC, 1979), moving $T_1N_1M_0$ from stage I to stage II and creates new subdivisions of stages III–IIIa and IIIb. Metastatic disease is now designated stage IV. Dillman *et al.* (1988) have reported on a trial (CALGB) in which 180 patients ($T_3$ and/or $N_2$, $M_0$) were randomised to radiotherapy (60 Gy in 6 weeks) alone or chemotherapy (cisplatin/vindesine) followed by radiotherapy.

Received 31 January 1989, accepted 9 February 1989.

Response or improvement was seen in 57% of patients receiving combined modality therapy compared with 40% of the radiotherapy alone patients ($P=0.06$). Estimated 1 year survival is 55% for the combined modality group and only 31% for the radiation group. Respective median survivals are 16.5 months and 8.5 months. Analysis of survival significantly favoured the combination arm (log rank $P=0.003$). Because the improvement in survival was greater than the stopping rules permitted the trial was discontinued early, the authors concluding that so-called 'protochemotherapy' improved survival.

Still other trials have examined the role of chemotherapy in combination with both surgery and radiotherapy. Lad *et al.* (1988) randomised 172 patients with incompletely resected lung cancer to either radiotherapy alone or radiotherapy plus cisplatin-based chemotherapy. There was a significantly longer recurrence-free interval for the chemotherapy group ($P=0.004$). Survival at 1 year was 14% greater in the chemotherapy arm than the control – this difference approaches statistical significance.

Advanced N-SCLC has been notoriously unresponsive to chemotherapy. Despite this, most randomised studies have failed to use a no-treatment control group preferring to compare different drug combinations (Simes, 1985). Previous clinical trials, of a decade or more ago, which used a no chemotherapy group, failed to substantiate any survival advantage for chemotherapy – the relevance of this observation is dubious since more active chemotherapy combinations have now been introduced. Two recent trials have compared cisplatin-based combinations with no chemotherapy. Woods *et al.* (1985) compared high dose cisplatin and vindesine with no chemotherapy – a study which has been updated recently (Williams *et al.*, 1988). Two hundred and one patients were randomly assigned to supportive care only or chemotherapy. The objective response rate for chemotherapy was 28% and the median duration of survival (27 weeks) was longer than that in the no chemotherapy group (17 weeks) though the differences were not significant ($P=0.33$). For patients with limited disease the corresponding median survivals were 45 and 26 weeks, the difference approaching statistical significance ($P=0.08$).

In a similar trial, Rapp *et al.* (1988) compared a policy of support alone with that of cisplatin, adriamycin, cyclophosphamide (CAP) or of cisplatin/vindesine (VP). One hundred and fifty patients were randomly allocated to one of the three arms. Response rates were CAP 15% and VP 25%. Median survivals were support only 17 weeks, CAP 25 weeks, VP 33 weeks. Survival in the VP arm was significantly greater than the control arm of best supportive care ($P<0.01$).

Both of these studies reported severe or life-threatening toxicity in a high proportion of patients so that the modest gains in survival (weeks at best) must be balanced against the unpleasantness of chemotherapy. Recently uncontrolled studies of new regimes continue to show even higher response rates but their effect on survival remains to be assessed (Cullen *et al.*, 1988). Unfortunately, none of the current studies has presented data on 'quality of life' so that the advantages of chemotherapy remain tenuous, being based solely on a small improvement (weeks) in survival and no chance of cure. The data from the surrogate study of MacKillop *et al.* (1986) suggests that most lung cancer experts do not feel that the small potential survival gain is worth the toxicity, since they would have refused consent to their own inclusion in chemotherapy trials in 69–91% of cases.

The reports discussed are highly selected and in general show positive results, but these must be weighed against many negative similar trials and a lack of information on the quality of life of patients receiving chemotherapy. Randomised clinical trials are starting to show that chemotherapy *can* improve survival, though the gains remain modest. Such small benefits must continue to be measured and weighed against the discomforts of chemotherapy. The advantages of chemotherapy remain debatable and it can only be recommended in the context of clinical trials. These trials should ideally continue to use an appropriate no chemotherapy control and should measure quality of life.

The simple answer to the question posed by Minna *et al.* (1982) is that chemotherapy should at present not be used as a routine therapy for non-small cell lung cancer. Until there is clear cut evidence of overall benefit from chemotherapy we should heed Will Roger's maxim:

It ain't what you don't know
that's the problem
It's what you think you
know that ain't so.

**References**

BRUNNER, K.W., MARTHALER, T. & MULLER, W. (1979). Adjuvant chemotherapy with cyclophosphamide (NSC-26271) for radically resected bronchogenic carcinoma: 9 year follow up. *Progr. Cancer Res. Ther.*, **11**, 411.

CULLEN, M.H., JOSHI, R., CHETCYAWARDANA, A.D. & WOODROFFE, C.M. (1988). Mitomycin, ifosfamide and cisplatin in non-small cell lung cancer: treatment good enough to compare. *Br. J. Cancer*, **58**, 359.

DILLMAN, R.O., SEAGREN, S.L., PROPERT, K. *et al.* (1988). Protochemotherapy improves survival in regional non-small cell lung cancer (NSCLC). *Proc. Am. Soc. Clin. Oncol.*, **7**, 195.

GRALLA, R.J., CASPER, E.S., KELSEN, D.P. *et al.* (1981). Cisplatin and vindesine combination chemotherapy for advanced carcinoma of the lung: a randomized trial investigating two dosage schedules. *Ann. Intern. Med.*, **95**, 414.

GRALLA, R.J., KRIB, M., BURKE, T. & MARTINI, N. (1987). Adjuvant chemotherapy. Fifth International Conference on the Adjuvant Therapy of Cancer. 11–14 March, Tucson, Arizona, p. 36.

HIGGINS, G.A. & SHIELDS, T.W. (1979). Experience of the Veterans Administration Surgical Adjuvant Group. *Progr. Cancer Res. Ther.*, **11**, 433.

HOLMES, E.C. & GAIL, M. (1986). Surgical adjuvant therapy for stage II and stage III adenocarcinoma and large cell undifferentiated carcinoma. *J. Clin. Oncol.*, **4**, 710.

LAD, T., RUBENSTEIN, L. & SADGHI, A. (1988). The benefit of adjuvant treatment for resected locally advanced non-small cell lung cancer. *J. Clin. Oncol.*, **6**, 9.

MACKILLOP, W.J., WARD, G.K. & O'SULLIVAN, B. (1986). The use of expert surrogates to evaluate clinical trials in non-small cell lung cancer. *Br. J. Cancer*, **54**, 661.

MINNA, J.D., HIGGINS, G.A. & GLATSTEIN, E.J. (1982). Cancer of the lung. In *Cancer: Principles and Practice of Oncology*, DeVita *et al.* (eds), p. 396. J.B. Lippincott: Philadelphia.

MOUNTAIN, C.F. (1986). A new international staging system for lung cancer. *Chest*, **89**, 225S.

MOUNTAIN, C.F., VINCENT, R.G. & SENLY, R. *et al.* (1979). A clinical trial of CCNU as surgical adjuvant treatment for patients with surgical stage I and stage II non-small cell lung cancer: preliminary findings. *Progr. Cancer Res. Ther.*, **11**, 421.

MOUNTAIN, C.M., GAIL, M.H. & LUNG CANCER STUDY GROUP (1981). Surgical adjuvant intrapleural BCG treatment for stage I non-small cell lung cancer. *J. Thorac. Cardiovasc. Surg.*, **82**, 649.

RAPP, E., PATER, J.L., WILLAN, A. *et al.* (1988). Chemotherapy can prolong survival in patients with advanced non-small cell lung cancer – report of a Canadian Multicentre randomized trial. *J. Clin. Oncol.*, **6**, 633.

SHIELDS, T.M., HIGGINS, G.A., HUMPHREY, E.W. *et al.* (1982). Prolonged intermittent adjuvant chemotherapy with CCNU and hydroxyurea after resection of carcinoma of the lung. *Cancer*, 1713.

SHIELDS, T.W., ROBINETTE, C.D. & KEEHN, M.S. (1974). Bronchial carcinoma treated by adjuvant cancer chemotherapy. *Arch. Surg.*, **109**, 329.

SIMES, R.J. (1985). Risk benefit relationships in cancer clinical trials: the ECOG experience in non-small cell lung cancer. *J. Clin. Oncol.*, **3**, 462.

AMERICAN JOINT COMMITTEE FOR CANCER (1979) *Staging of Lung Cancer. Staging and End Results Reporting: Task for on Lung Cancer*. AJCC: Chicago, IL.

VAN HOUTTE, P., ROCMANS, P., SMETS, P. *et al.* (1980). Postoperative radiation therapy in lung cancer. A controlled trial after resection of curative design. *Int. J. Radiat. Oncol. Biol. Phys.*, **6**, 983.

WILLIAMS, C.J., WOODS, R., LEVI, J. & PAGE, J. (1988). Chemotherapy for non-small cell lung cancer: a randomised trial of cisplatin/vindesine vs. no chemotherapy. Perugia International Cancer Conference 1: Chemotherapy of non-small cell lung cancer – current status. Perugia, 17–18 June, p. 43.

WOODS, R.L., LEVI, J.A., PAGE, J. *et al.* (1985). Non-small cell cancer: a randomized comparison of chemotherapy with no chemotherapy. *Proc. Am. Soc. Clin. Oncol.*, **4**, 177.